# Prevalence and molecular characterization of *Clostridium difficile* isolated from European Barn Swallows (*Hirundo rustica*) during migration

**DOI:** 10.1186/1746-6148-10-40

**Published:** 2014-02-08

**Authors:** Petra Bandelj, Tomi Trilar, Rok Blagus, Matjaz Ocepek, Joyce Rousseau, J Scott Weese, Modest Vengust

**Affiliations:** 1 Veterinary faculty, University of Ljubljana, Ljubljana SI-1115, Slovenia; 2 Slovenian Museum of Natural History, Ljubljana SI-1000, Slovenia; 3 Institute for Biostatistics and Medical Informatics, University of Ljubljana, Ljubljana SI-1104, Slovenia; 4 Department of Pathobiology, Ontario Veterinary College, University of Guelph, Guelph, Ontario N1G 2W1, Canada

**Keywords:** *Clostridium difficile* infection, Zoonosis, Migrating passerines, Birds, Cattle farming

## Abstract

**Background:**

*Clostridium difficile* is an important bacterial pathogen of humans and a variety of animal species. Birds, especially migratory passerine species, can play a role in the spread of many pathogens, including *Clostridium difficile*. Barn Swallows (*Hirundo rustica*) nest in close proximity to human habitats and their biology is closely associated with cattle farming. Therefore, we hypothesized that Barn Swallows can be the reservoir of *Clostridium difficile*.

**Results:**

Barn Swallows (n = 175) were captured on their autumn migration across Europe to sub-Saharan Africa. Droppings were collected from juvenile (n = 152) and adult birds (n = 23). Overall prevalence of *Clostridium difficile* was 4% (7/175); 4.6% (7/152) in juvenile birds and 0/23 in adults. *Clostridium difficile* ribotypes 078, 002 and 014 were identified, which are commonly found in farm animals and humans. Three new *Clostridium difficile* ribotypes were also identified: SB3, SB159 and SB166, one of which was toxigenic, harbouring genes for toxins A and B.

**Conclusions:**

Results of this study indicate that Barn Swallows might play a role in national and international dissemination of *Clostridium difficile* and could serve as a source for human and animal infection. *Clostridium difficile* ribotype 078 was identified, which has been reported as an emerging cause of community-associated *Clostridium difficile* infection in humans. Based on this and other studies, however, it is more likely that Barn Swallows have a more indicative than perpetuating role in *Clostridium difficile* epidemiology.

## Background

*Clostridium difficile* (CD) is present in almost all human environments and is a potential zoonotic pathogen that can be isolated from a variety of animal species [1-5]. It is the most commonly diagnosed cause of antimicrobial- and hospital-associated diarrhoea [6] and is an emerging cause of community-associated disease [7,8]. The presence of CD in animals, including those that come in close contact with humans as pets or through the food chain [1,9], and the significant overlap between the types of CD isolated from humans and animals [10-12], has lead to suggestion that CD might be a zoonotic and foodborne pathogen [9,12].

Recognition of different potential sources of CD transmission remains a pressing clinical and investigative quest. Migratory birds travel between continents twice each year and are responsible for the transmission of several pathogens [13] and/or disease vectors [14]. The world’s largest bird migration system is the Palaearctic–African flyway, which involves an estimated 2 billion passerines and near-passerines migrating between the European continent and sub-Saharan Africa [15].

An epidemiological study on mostly migrating passerine birds in Europe suggested that they are unlikely to serve as a carrier or reservoir of CD [16]. However, the study [16] was conducted in the region where Barn Swallows (*Hirundo rustica*) were not congregating during their migration south, and included only garden birds unlikely to dwell in habitats intensively cultivated by humans. On the other hand, Barn Swallows, come in close contact with human habitats [17,18], as do House Sparrows (*Passer domesticus*), which are a non-migrating passerine bird, that have been associated with CD when sampled on pig farms [19].

Barn Swallows are among most common and widespread migratory birds in the Palaearctic. In Europe they most commonly nest in cattle farms inside the barns or stables, which had a significant evolutionary effect on Barn Swallow biology with regards to their nesting habits. Any changes in their environment significantly affect their population [17,18]. Its habit of nesting in buildings associated with human habitation has made this species one of the most familiar bird species in the world [20] as well as a potential source for international and local CD perpetuation. The aims of the present study were to determine the prevalence of CD in Barn Swallows, and to characterize CD isolates.

## Methods

Sampling of Barn Swallows was conducted once in September 2011 in the central part of Slovenia (46° 0′ 2.12″ N, 14° 22′ 58.27″ E) in an area identified as a Barn Swallow congregation point during their autumn migration across Europe (average week temperature 21.8°C, average week humidity 69.5%). One hundred seventy-five (n = 175) Barn Swallows were captured with mist nets and placed individually in clean custom made hot pressed plastic containers with air holes. Bird faeces, if present, were collected from containers using sterile gloves (Ansell Ltd, UK) after the birds were removed for standard general inspection, ringing and release to continue with their migration. Faeces were transferred into 2 mL sterile tubes (Eppendorf Tubes, Germany) and stored at -20°C until analysed for the presence of CD spores.

The study was carried out with animal ethics approval by the Ministry of the Environment and Spatial Planning (document No.: 35601-10/2010-6).

### *Clostridium difficile* culture

Entire samples were inoculated into 9 mL of CD moxalactam norfloxacin (CDMN) enrichment broth (Oxoid Ltd; Nepean, ON Canada) containing 0.1% sodium taurocholate. Anaerobic incubation at 37°C lasted for 7 days. An aliquot of broth was transferred to a new vial and an equal amount of anhydrous alcohol was added to each sample. This was followed by a 60 min incubation at room temperature, centrifugation (3800 g for 10 min) and inoculation of the pellets onto CDMN (Oxoid Ltd; Nepean, ON Canada) agar. Further incubation in an anaerobic chamber for 2 days at 37°C was followed by another 3 days if necessary. Isolation and identification of CD was based on the characteristic morphology and odour of the colonies, Gram stain and the presence of the L-proline aminopeptidase activity (Remel Inc, Lenexa, KS, USA). One single colony for each isolate was subcultured and stored at -80°C and re-cultured prior to molecular analysis.

### Molecular analysis

Extraction of DNA was done on pure cultures after they were obtained from blood agar with a Chelex resin-based DNA extraction commercial kit (InstaGene Matrix, Bio-Rad Laboratories, USA), following the manufacturer’s instructions. Extracted DNA was used as template for further molecular analysis. Ribotyping was performed as previously described by [21]. Ribotpyes were assigned visually based on comparison with an internal library of ribotypes as well as reference strains from the Cardiff ECDC reference library. Testing for genes encoding toxins A (*tcdA*) and B (*tcdB*) was performed by PCR as previously described by [22]. The presence of the gene encoding the binding component of the binary toxin (*cdtA*) was detected as described in [23].

## Results

A total of 175 samples were taken from 152 juvenile and 23 adult Barn Swallows. Seven (7/175; 4%) samples were positive for CD; 7/152 (4.61%) juveniles and 0/23 adults. Five (5/7; 71%) isolates were toxigenic. All five toxigenic isolates possessed *tcdA* and *tcdB* while two also possessed *cdtA. Clostridium difficile* isolates that were toxin A, B and CDT (*Clostridium difficile* toxin/binary toxin) positive, were identified as ribotype 078. Single isolates of ribotypes 002 and 014 were also identified. The remaining three ribotypes, one of which was toxigenic, had not been previously identified in this laboratory or documented in the available Cardiff ECDC collection (Table 1).

**Table 1:** ***Clostridium difficile *** **toxins and ribotypes in Barn Swallows ( *Hirundo rustica *)**

***Clostridium difficile***	
**Toxins**	**Ribotype**
A + B + CDT+	078
A + B + CDT-	002
A + B + CDT-	014
A + B + CDT-	SB166*
A-B-CDT-	SB3*
A-B-CDT-	SB159*

*Newly identified ribotypes.

## Discussion

This is the first study investigating Barn Swallow as a possible source of CD for farm animals and humans. In the past, there has been increasing concern about animals as potential CD reservoirs and sources of human exposure [12,24]. Of particular note is the finding of ribotype 078. This strain is common in food animals [4,25,26] and has been reported as an emerging and increasing cause of community-associated *Clostridium difficile* infection (CDI) in humans [25,27,28]. Two of the three other toxigenic ribotypes identified in this study (002, 014) can be found both in humans and animals [26,29]. Three new CD ribotypes were also identified (SB3, SB159, SB166), one possessing A + B + CDT- (SB166).

It was interesting that CD was only found in juvenile birds. The sample population in this study was predominantly juvenile birds, which is in concordance with the expected ratio of juvenile birds on migration (>80%) [16,30]. In most studied animal species, CD tends to predominate in younger animals [31,32]. There is no evidence that CD causes disease in Barn Swallows.

Currently, the epidemiology of community-associated CDI is poorly understood. Food animals and food have been suggested as sources of human exposure [9,33]; however, other potential forms of exposure in the community must be considered. While the high prevalence of CD in some farm animal groups clearly indicates that they could be reservoirs of CD, the potential for other animals to act as a source of CD from farms to the broader human or animal population is intriguing. The biology of Barn Swallows potentially makes them a very efficient vector for CD dissemination. They are the most common and widespread migratory bird in Europe and cohabit with farm animals and humans [17,18,20].

Passerine birds were previously not determined to be the source of CD; however, only passerines unlikely to come in close contact with humans have been investigated [16]. In contrary, House Sparrows, a non-migrating passerine, had previously been associated with CD on pig farms in the Netherlands [19]. European Barn Swallows, the species studied here, preferably nest within the cattle barns during warm months of the year, and spend the winter in Sub-Saharan Africa. Some can migrate to Arabia and to the Indian sub-continent [17,18]. In our study, a total of 4% of all captured and sampled Barn Swallows were positive for CD, which reflects the prevalence of adult cattle in some reports. Given the presence of CD in the bovine population, with reported prevalence of 2.4-6.3% in adult cattle and 7.6-51% in calves [3,5,34] and the nesting locations of Barn Swallows, it is certainly plausible that these birds could acquire CD on farms. Interestingly, to date, including this study, CD was isolated only from wild birds that are associated with intensively farmed habitats [16,19]. Therefore, it is more likely that Barn Swallows have a more indicative than perpetuating role in CD epidemiology.

## Conclusions

Results of this study showed that Barn Swallows could potentially be a source of CD in humans and animals. During their congregation at migration destinations inter-individual and interspecies horizontal transmission can occur [13]. However, based on this and previous studies on the prevalence of CD in wild passerine birds [16,19], and studies associated with farm animals and humans [10,27,31,32,35], Barn Swallows may more realistically be an indicator and not the source of the contamination of CD in the environment. It seems that intensive farming and hospital environment are the sources of CD, and only humans and/or animals associated with such environment perpetuate CD.

## Abbreviations

CD: *Clostridium difficile*; CDI: *Clostridium difficile* infection.

## Competing interests

Authors have no competing interests to declare.

## Authors’ contributions

All of the authors contributed to the conception design, interpretation of data, drafting the article and critical revision of the manuscript. PB, TT and MV collected samples from Barn Swallows. JSW and JR performed bacterial culture and molecular analysis. All authors approved the final version of this manuscript.
